# Analysis of the Plasticity of Circulating Tumor Cells Reveals Differentially Regulated Kinases During the Suspension‐to‐Adherent Transition

**DOI:** 10.1002/cam4.70339

**Published:** 2024-10-18

**Authors:** Daniel J. Smit, Konstantin Hoffer, Bettina Bettin, Malte Kriegs, Laure Cayrefourcq, Udo Schumacher, Klaus Pantel, Catherine Alix‐Panabières, Manfred Jücker

**Affiliations:** ^1^ Institute of Biochemistry and Signal Transduction University Medical Center Hamburg‐Eppendorf Hamburg Germany; ^2^ Institute of Tumor Biology University Medical Center Hamburg‐Eppendorf Hamburg Germany; ^3^ Department of Radiotherapy & Radiation Oncology University Medical Center Hamburg‐Eppendorf Hamburg Germany; ^4^ Laboratory of Rare Human Circulating Cells (LCCRH) University Medical Center of Montpellier Montpellier France; ^5^ CREEC/CANECEV, MIVEGEC (CREES) University of Montpellier, CNRS, IRD Montpellier France; ^6^ European Liquid Biopsy Society (ELBS) Hamburg Germany; ^7^ Institute of Anatomy and Experimental Morphology University Medical Center Hamburg‐Eppendorf Hamburg Germany; ^8^ Medical School Berlin Berlin Germany

**Keywords:** cellular plasticity, circulating tumor cells, functional kinome profiling, signal transduction

## Abstract

**Background:**

Research on circulating tumor cells (CTCs) offers the opportunity to better understand the initial steps of blood‐borne metastasis as main cause of cancer‐related deaths. Here, we have used the colon cancer CTC‐MCC‐41 and breast cancer CTC‐ITB‐01 lines, which were both established from human CTCs as permanent cell lines as models to further study CTC biology with special emphasis on anchorage‐independent survival and growth.

**Methods and Results:**

Both cell lines showed a marked intrinsic plasticity to switch between suspension and adherent in vitro growth, in 2D adherent culture conditions, and established an equilibrium of both growth patterns with predominant adherent cells in the CTC‐MCC‐41 line (77%) and suspension cells in the CTC‐ITB‐01 line (85%). Western blot analysis revealed a higher expression of pERK1/2 in CTC‐ITB‐01 adherent cells compared to the suspension counterpart that suggested the involvement of kinases in this process. Subsequent functional kinome profiling identified several serine/threonine as well as tyrosine kinases that were differentially regulated in adherent and suspension CTCs. In the adherent cells of the breast cancer line CTC‐ITB‐01 the activity of MSK1, Src family kinases and the PKG family was increased compared to the suspension counterpart. In adherent cells of the colorectal CTC‐MCC‐41 line, an increased activity of TYRO3 and JAK2 was detected, whereas p38 MAPK was strongly impaired in the suspension CTC‐MCC‐41 cells. Some of the regulated kinases, which include the Src family, TYRO3, MSK1, JAK2 and p38 MAPK, have been associated with crucial cellular processes including proliferation, migration and dormancy in the past.

**Conclusions:**

The investigated CTC lines exhibit a high plasticity, similar to the concept of ‘adherent‐to‐suspension transition (AST)’ that was recently suggested as a new hallmark of tumor biology by Huh et al. Moreover, we identified differentially regulated kinome profiles that may represent potential targets for future studies on therapeutic interventions.

## 1 Introduction

Over the past years, circulating tumor cells (CTCs) gained tremendous attention in cancer research. CTC lines opening up new avenues of understanding CTC biology and the molecular characteristics of the metastasis‐competent cells [1]. As metastasis is the main course of cancer‐associated deaths, it is of utmost importance to understand the underlying mechanisms. In contrast to short term cultivation of patient derived CTCs, the stable cell lines enable basic research and provide a precious model for studying CTC biology [2]. Signal transduction pathways including PI3K/AKT/mTOR and RAS/RAF/MEK/ERK signaling as well as other pathways play a crucial oncogenic role in the development and progression of cancer [3, 4]. Several proteins in these pathways belong to the family of kinases, which can be further divided into serine/threonine or tyrosine kinases, depending on their target residues [5]. In our previous study using the stable colorectal cancer (CRC) CTC line referred to as ‘CTC‐MCC‐41’ [6] we revealed a strong activation of the PI3K/AKT/mTOR signaling pathway as well as a high susceptibility towards dual AKT/mTOR inhibition underlining the importance of this signaling pathway in this cell line for proliferation [7]. In the past, a distinct molecular as well as metabolic profile has been reported for the CTC‐MCC‐41 line, that was not present in established CRC cell lines (e.g., HT29) [8]. Moreover, the CTC‐MCC‐41 cell line features an intermediate epithelial‐mesenchymal phenotype with certain stem cell associated molecular traits that has also been reported for other long‐term stable CTC lines, for example, in breast cancer [9]. Of note, the CTC‐ITB‐01 cell line, a permanent CTC line derived from a breast cancer patient, retained the molecular characteristics of the primary tumor and thereby provides a valuable model for studying the tumor biology of CTCs [9]. Interestingly, the distinct growth pattern with presence of both, adherent and suspension cells in the same culture, was reported in the past in other CTC lines [10, 11]. A recent study by Huh et al, coined the term ‘adherent‐to‐suspension transition’ that refers to the reprogramming of adherent cells into suspension cells [12] that is independent of the commonly studies epithelial mesenchymal transition (EMT) that has been strongly associated with metastasis [13]. In this study, we investigate the molecular characteristics of suspension and adherent cells with respect to their signal transduction pathway activation and kinome activation in two unique long‐term human CTC lines (CTC‐MCC‐41 and CTC‐ITB‐01). We demonstrate that several kinases are differentially regulated in suspension versus adherent cells of the CTC lines.

## 2 Material And Methods

### 2.1 Standard Cell Culture

The CTC‐MCC‐41 cells were cultivated in RPMI Medium 1640—GlutaMAX‐I (Thermo Fisher Scientific, Waltham, Massachusetts, USA) with 10% fetal calf serum (FCS), 1% penicillin/streptomycin (P/S) and 5 mL insulin‐transferrin‐supplement A. The media was supplemented with human epidermal growth factor (50 ng/mL), fibroblast growth factor 2 (10 ng/mL) and hydrocortisone (0.1 μg/mL). For CTC‐ITB‐01 cells, choleratoxin (0.2 μg/mL) was added to the abovementioned media. Adherent CTCs were detached using accutase cell detachment solution (Capricorn Scientific, Ebsdorfergrund, Germany). When cultured in 2D adherent conditions, the CTCs grow as adherent and suspension cells at the same time, so fractions were separately treated and pooled afterwards. The CTC lines were cultivated in a cell culture incubator (Heraeus HeraCell, Hanau, Germany) at 37°C, 5% CO_2_. MCF‐7 cells were cultured in DMEM medium (Thermo Fisher Scientific, Waltham, Massachusetts, USA) supplemented with 10% FCS and 1% P/S. Cells were detached using Trypsin 0.05% EDTA solution (Life Technologies, Carlsbad, California, USA). All cells were regularly tested for mycoplasma contamination. Light microscopy was conducted using an inverted light microscope at 10× magnification (Carl Zeiss, Oberkochen, Germany).

### 2.2 Cell Counting Assays

Cells derived from the cell culture flask under routine cultivation as outlined in section 2.1 were used at approximately 60% confluence. The suspension and adherent cell fractions of the CTC‐MCC‐41 and CTC‐ITB‐01 line were separated as following: The supernatant was collected to retrieve the suspension cells and transferred to a falcon tube. Cells were centrifuged, washed with PBS, centrifuged again and resuspended in cell culture media. In order to retrieve the adherent fraction, the cell culture flask was washed with PBS and thereafter the remaining adherent cells were detached using accutase cell detachment solution, centrifuged, washed with PBS, centrifuged again and resuspended in cell culture media.

An aliquot of the well‐mixed cell solution was diluted in trypan blue solution (1:1) prior to cell counting. Automatic cell counting was performed using the Countess II FL (Thermo Fisher Scientific, Waltham, Massachusetts, USA) according to the manufacturer's instructions. Cell counts of alive cells were considered for analysis and used to calculate the distribution of adherent and suspension cells in Section 3.1.

The counted cells were then plated from each of the fraction (i.e., suspension only or adherent only) at the same cell count (5 × 10^4^ cells) in well plates. Forty‐eight hours later, the cells were retrieved from the well plates using the same procedure as outlined above and counted again. The percentage in relation to the original number of suspension or adherent cells seeded before was calculated and referred to as the ‘restauration ratio’.

### 2.3 SDS‐PAGE Electrophoresis and Western Blot Analysis

NP40‐lysates from whole cells were prepared as described previously [7]. Protein concentration was determined by lowry‐assay. Proteins were separated by SDS page gel electrophoresis and transferred to a 0.45 μm nitrocellulose membrane. Afterwards the membrane was incubated with primary antibodies against mTOR, pmTOR (S2448), AKT, pAKT (S473), ERK1/2, pERK1/2 (T202/Y204), S6, pS6 (S240/S244), pMSK1 (S376), pPKG2 (S126) (Biorbyt Ltd., Cambridge, United Kingdom) pSRC family (Y416), pJAK2 (Y1007/Y1008), pTYRO3 (Y681), pGSK‐3α/β (S21/S9), pp38 MAPK (T180/Y182), p54 JNK (T183/Y185), p46 JNK (T183/Y185) (unless indicated otherwise all purchased from Cell Signaling Technology, Danvers, MA, USA). Appropriate species specific HRP‐linked secondary antibodies against rabbit IgG or mouse IgG were purchased from Cell Signaling Technology (Danvers, MA, USA). Equal protein loading was confirmed by Ponceau S staining (Serva Electrophoresis GmbH, Heidelberg, Germany) as well as housekeeping protein HSC70 (Santa Cruz Biotechnology Inc., Dallas, TX, USA). A complete list of antibodies used in this study is available in the supporting information (Table S1). Western blot images were acquired using the ImageQuant LAS 4000 system (GE Healthcare Bio‐Sciences, Pittsburgh, PA, USA). Densitometric quantification of the western blots was performed using the AIDA Image Analysis software (Elysia‐raytest GmbH, Straubenhardt, Germany).

### 2.4 Functional Kinome Profiling

Whole cell lysates from CTC‐MCC‐41 and CTC‐ITB‐01 suspension and adherent fraction were prepared on ice in mammalian protein extraction reagent (M‐PER buffer) (Thermo Fisher Scientific, Waltham, Massachusetts, USA) supplemented with Halt Phosphatase Inhibitor and EDTA‐free Halt Protease Inhibitor Cocktail (Pierce Biotechnology Inc., Rockford, IL, USA). Thereafter, functional kinome profiling for serine/threonine as well as tyrosine kinases was performed using the PamStation 12 using the PamChip4 (both PamGene International, ‘s‐Hertogenbosch, the Netherlands). For serine/threonine kinase profiling 1 μg and for tyrosine kinase profiling 5 μg of the protein lysate was used. Analysis and visualization were performed using the BioNavigator software version 6.3.67.0 (PamGene International, ‘s‐Hertogenbosch, the Netherlands).

### 2.5 Statistical Analysis and Visualization

Statistical analysis and visualization were performed with GraphPad Prism 9.4.1 (GraphPad Software Inc., San Diego, CA, USA). A *p* value < 0.05 was considered as statistically significant. *p* values were encoded into asterisks (ns *p* > 0.05; *p ≤ 0.05; **p ≤ 0.01; ***p ≤ 0.001; ****p ≤ 0.0001).

## 3 Results

### 3.1 Growth Pattern of CTCs in Culture

**FIGURE 1:**
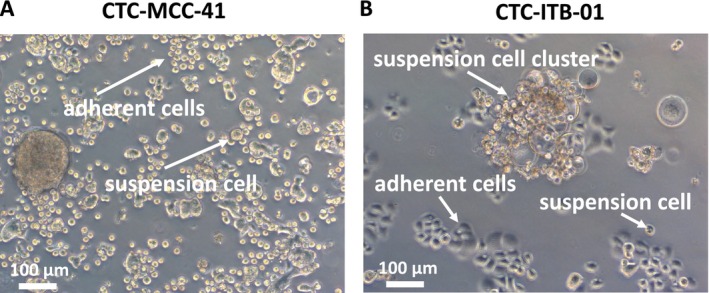
The CTC‐MCC‐41 and CTC‐ITB‐01 lines grow in suspension and adherent in regular cell culture. Representative light microscopy image of CTC‐MCC‐41 (A) and CTC‐ITB‐01 (B) suspension and adherent cell morphology with annotations. The scale bar in both pictures represents 100 μm.

Colorectal cancer CTC line CTC‐MCC‐41 and breast cancer CTC line CTC‐ITB‐01 demonstrate a distinct growth pattern in 2D adherent cell culture. In comparison to immortalized breast cancer or colorectal cell lines, the CTCs grow in an adherent and suspension fraction at the same time. The CTC‐MCC‐41 cell line consists of small round cells in the adherent fraction and similar cell morphology in the suspension fraction. Less frequently, larger tumor cells or larger CTC clusters of more than one cell can be observed (Figure 1A). The adherent CTC‐ITB‐01 cells have an epithelial cell morphology with a small cytoplasm. CTC‐ITB‐01 suspension cells are of a round shape and mostly organized in larger cell clusters (Figure 1B).

**FIGURE 2:**
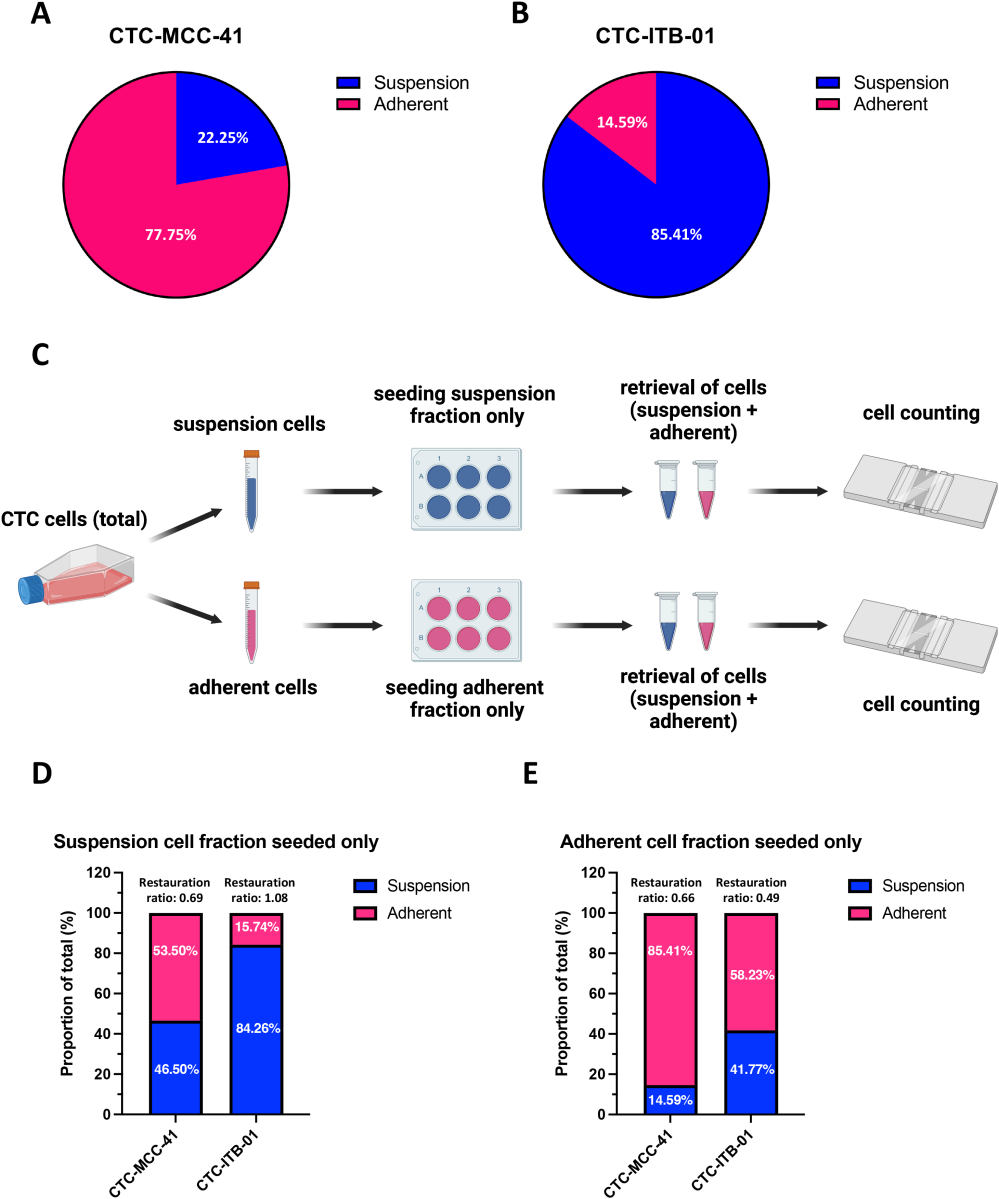
Analysis of suspension and adherent fractions in CTC‐MCC‐41 and CTC‐ITB‐01 cell lines and their capacity to switch their phenotype. (A) Proportion of CTC‐MCC‐41 suspension and adherent cells in relation to the total cell count in 2D culture. Data were derived from cell counting experiments (*n* = 4). (B) Proportion of CTC‐ITB‐01 suspension and adherent cells in relation to the total cell count under normal cell culture conditions. Data were derived from cell counting experiments (*n* = 3). (C) Experiment setup to evaluate the capacity to give rise to the respective counterpart (i.e., adherent or suspension) after seeding of only one of the fractions. The Figure was created using BioRender.com. (D) Proportion of CTC‐MCC‐41 and CTC‐ITB‐01 suspension and adherent cells in relation to the total cell count (including its restauration ratio) after seeding of only the suspension cell fraction (*n* = 6). (E) Proportion of CTC‐MCC‐41 and CTC‐ITB‐01 suspension and adherent cells in relation to the total cell count (including its restauration ratio) after seeding of only the adherent cell fraction (*n* = 3). Cells were counted 48 h after plating.

Analysis of the adherent and suspension fractions under normal cell culture conditions revealed a higher fraction of adherent cells (77.75%) compared to suspension cells (22.25%) for the CTC‐MCC‐41 cell line (Figure 2A). In contrast, in the CTC‐ITB‐01 cell line only 14.59% of the cells were in the adherent fraction versus 85.41% in the suspension fraction (Figure 2B). In the CTC‐ITB‐01 cell line, the large suspension cell clusters are mainly responsible for this ratio.

In a next step, to evaluate the plasticity of the cell lines to become suspension cells or adherent cells and vice versa, the fractions were separated and only one of the fractions (i.e., suspension or adherent) was seeded (Figure 2C). Thereafter, the capacity of the cell line to give rise to their respective counterpart that is, adherent cells for suspension fraction and vice versa was assessed by cell counting (Figure 2D,E). Interestingly, both cell lines had the capacity to form the respective counterpart after overnight cultivation in the incubator. CTC‐MCC‐41 are capable to almost restore the original ratio of adherent to suspension cells that is, 77.8%–22.3% respectively after plating only the suspension fraction (53.5% adherent cells and 46.5% suspension cells, restauration ratio 0.69) indicating a high plasticity of these CTCs. Interestingly, seeding only the adherent fraction of CTC‐MCC‐41 cells, a lower amount of suspension cells (14.59%) was observed compared to the initial ratio in the standard cell culture (restauration ratio 0.66). Regarding the CTC‐ITB‐01 cell line, a complete restauration of the initial proportions of suspension and adherent cells (84.26% and 15.74%, respectively) was observed after only seeding the suspension cells (restauration ratio 1.08). Similar to the other CTC line, the capability to restore the initial proportions (i.e., predominant suspension phenotype) within the normal cell culture was impaired when only plating the adherent cells in CTC‐ITB‐01 cells.

### 3.2 Signaling Pathway Analysis of Suspension and Adherent Cells

To understand the underlying mechanism of the phenotypic behavior of the CTCs in the 2D cell culture, western blot analysis was performed. In CTC‐MCC‐41 cells, a significant higher expression of pAKT S473 in adherent cells was detected in previous work [7]. Regarding pmTORC1 S2448, downstream substrate pS6 S240/S244 and pERK1/2 T202/Y204 no significant difference was observed comparing suspension and adherent CTCs [7].

**FIGURE 3:**
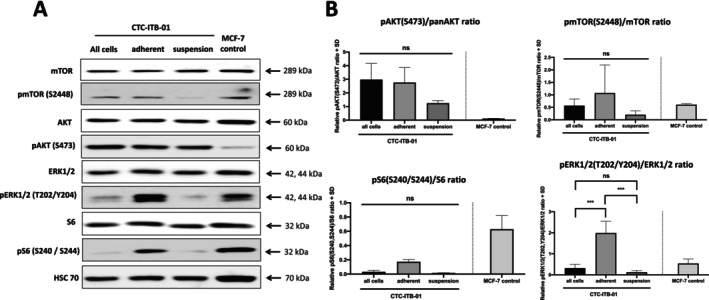
Signaling pathway analysis of CTC‐ITB‐01 suspension and adherent fraction. (A) Western blot analysis of CTC‐ITB‐01 adherent cells, suspension cells, the whole cell fraction of CTC‐ITB‐01 and MCF‐7 cell line regarding PI3K/AKT/mTOR and RAS/RAF/MEK/ERK signaling activity. (B) Densitometric quantification of the pAKT(S473)/AKT ratio, pmTOR(S2448)/mTOR ratio, pS6(S240/244)/S6 ratio and pERK1/2(T202/Y204)/ERK1/2 ratio (*n* = 3, one‐way ANOVA with Tukey's post hoc test (ns *p* > 0.05, ***p ≤ 0.001, mean values with standard deviation).

CTC‐ITB‐01 cells were also separated in the adherent and suspension fraction and subjected to western blot analysis (Figure 3A). Like the colorectal CTCs, an increased, but not statistically significant, expression of pAKT S473 was observed in adherent CTC‐ITB‐01 cells compared to suspension cells (*p* = 0.1502). Moreover, an increased expression of pS6 S240/S244 was observed in adherent cells compared to suspension fraction (*p* = 0.2288). Interestingly, a strong and statistically significant higher expression of pERK1/2 T202/Y204 was observed in adherent CTC‐ITB‐01 cells compared to suspension cells (*p* = 0.0003) (Figure 3B).

### 3.3 Functional Kinome Profiling of Suspension and Adherent CTCs

**FIGURE 4:**
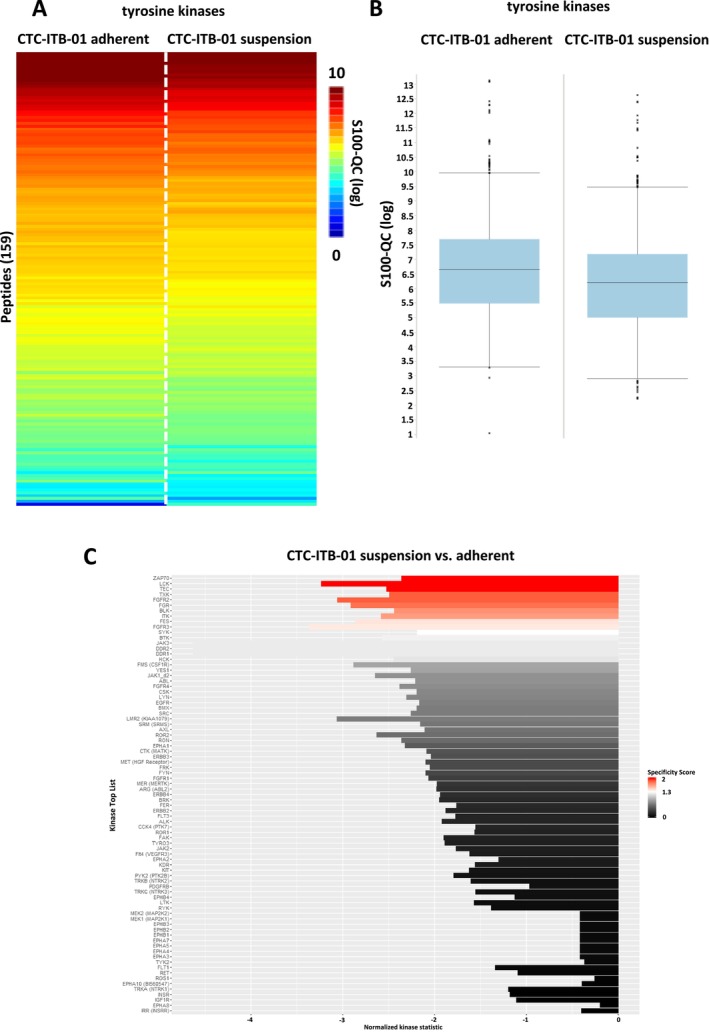
Tyrosine kinase profiling in CTC‐ITB‐01 suspension and adherent fraction. (A) Heatmap of mean log‐transformed S100‐QC values from tyrosine kinase activity measurement of CTC‐ITB‐01 adherent and suspension cells (*n* = 3 each group). (B) Boxplots of mean log‐transformed S100‐QC values (*n* = 3 each group) from tyrosine kinase analysis of CTC‐ITB‐01 adherent and suspension cells. (C) Predicted upstream tyrosine kinase list based on the results from the PamGene array (196 tyrosine peptides). Significant regulated upstream kinases were color coded based on their specificity score (white [1.3] to red [2]). Non‐significant upstream kinases are colored in a gray to black gradient.

In a next step, kinome profiling of adherent and suspension fractions of both CTC lines was performed. In general, breast cancer CTC line CTC‐ITB‐01 suspension and adherent cells showed a high degree of homology as indicated by boxplots and heatmaps with respect to tyrosine kinases (Figure 4A,B). Among the tyrosine kinases, 11 kinases were significantly upregulated in the adherent fraction of CTC‐ITB‐01 compared to the suspension fraction. No upregulation of tyrosine kinases was detected in the CTC‐ITB‐01 suspension cell fractions (Figure 4C). Among the identified tyrosine kinases, several Src kinase family members including LCK, FGR, BLK were upregulated in adherent cells. Additionally, FGF signaling associated receptor tyrosine kinases including FGFR2 and FGFR3 were detected (Figure 4C).

**FIGURE 5:**
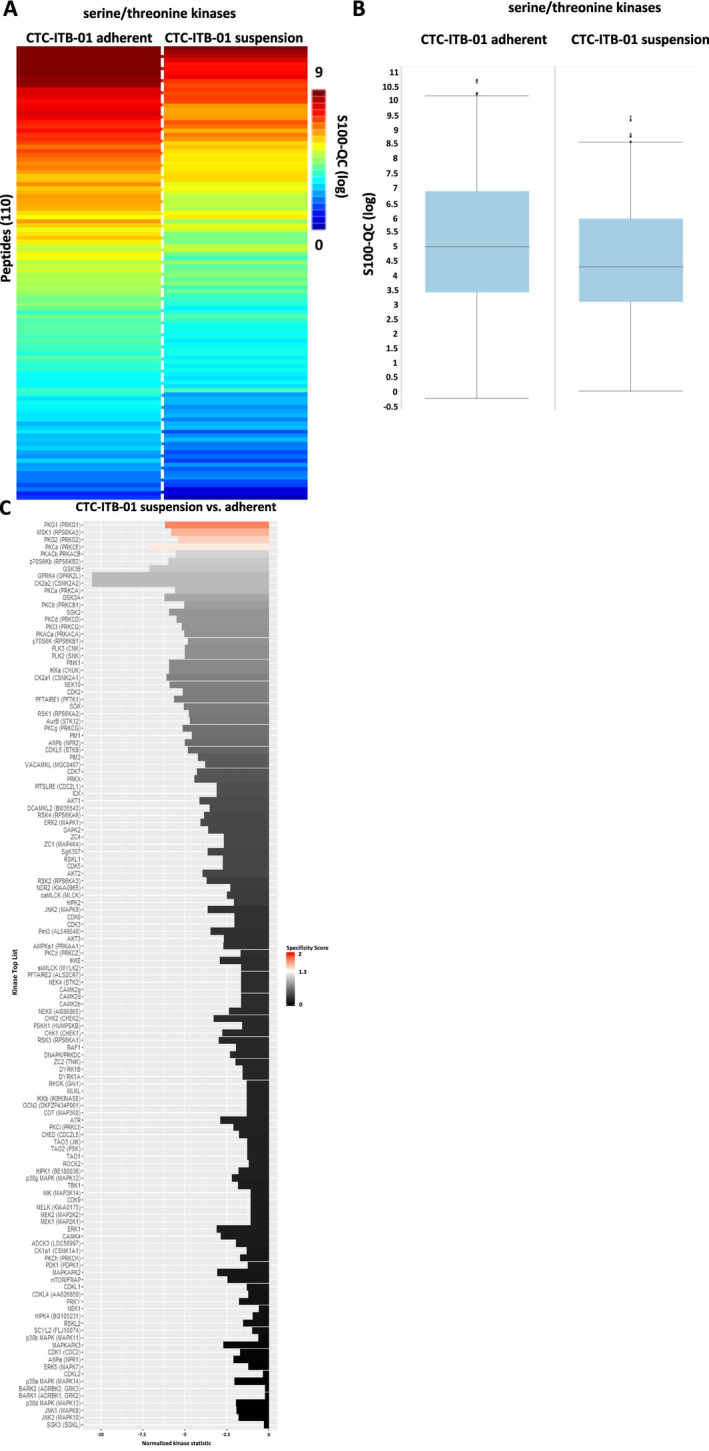
Serine/threonine kinase profiling in CTC‐ITB‐01 suspension and adherent fraction. (A) Heatmap of mean log‐transformed S100‐QC values from serine/threonine kinase activity measurement of CTC‐ITB‐01 adherent and suspension cells (*n* = 3 each group). (B) Boxplots of mean log‐transformed S100‐QC values (*n* = 3 each group) from serine/threonine kinase analysis of CTC‐ITB‐01 adherent and suspension cells. (C) Predicted upstream serine/threonine kinase list based on the results from the PamGene array (144 serine/threonine peptides). Significant regulated upstream kinases were color coded based on their specificity score (white [1.3] to red [2]). Non‐significant upstream kinases are colored in a gray to black gradient.

Analysis of serine/threonine kinases (Figure 5) showed an upregulation of four serine/threonine kinases including the protein kinase G family (PKG1 and PKG2, respectively), PKCε and MSK1 in adherent cells of the CTC‐ITB‐01 line (Figure 5C).

**FIGURE 6:**
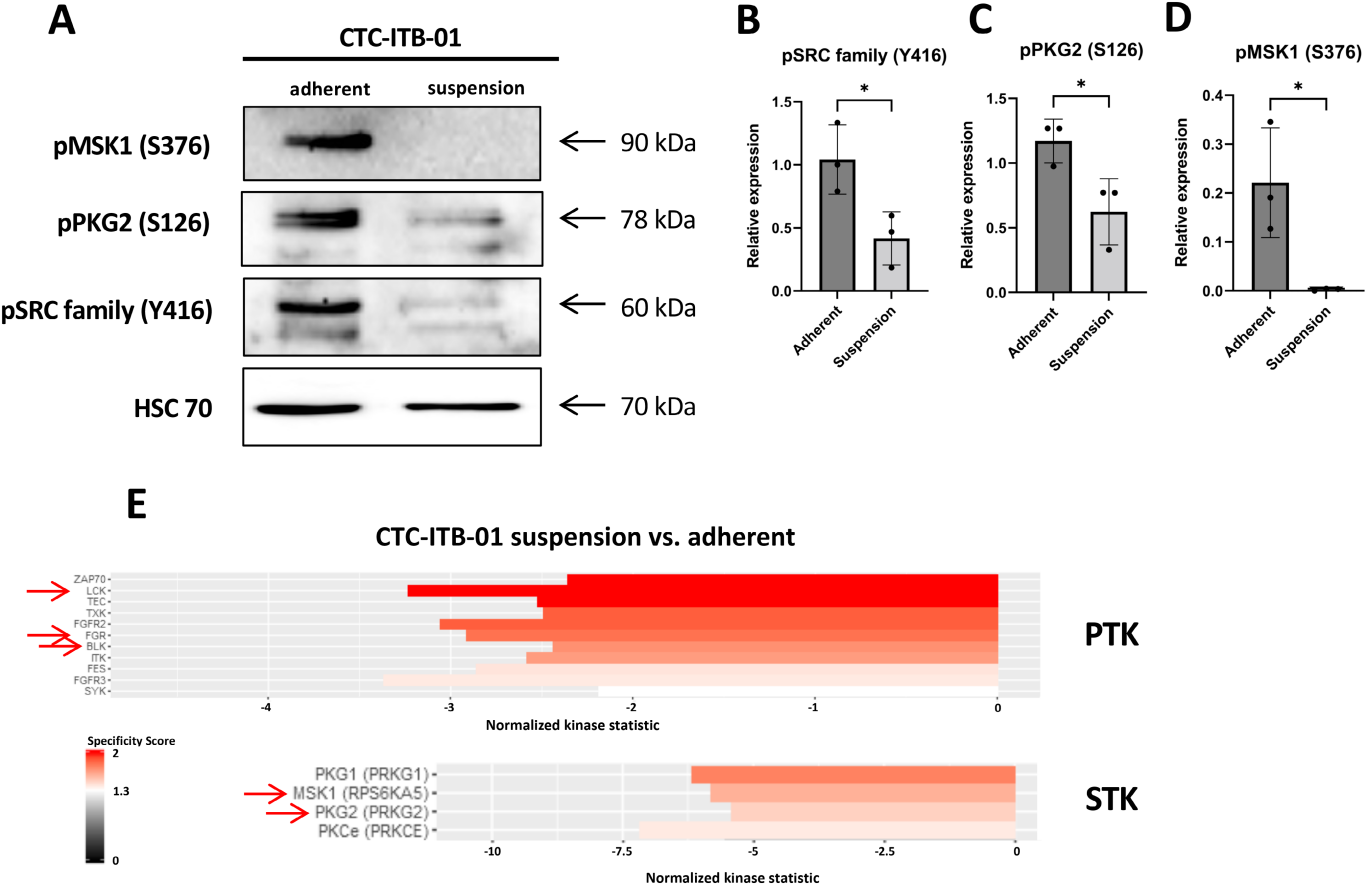
Confirmation of identified kinases in CTC‐ITB‐01 cells by western blot analysis. (A) Western blot analysis of predicted upstream kinases by the PamGene arrays in CTC‐ITB‐01 adherent and suspension cells fraction. The expression of phosphorylated SRC family (Y416) (B), PKG2 (S126) (C) and pMSK1 (S376) (D) was used to confirm the results from the kinome profiling (*n* = 3, unpaired two‐sided *t*‐test, *p ≤ 0.05, mean values with standard deviation). (E) Excerpt from the kinase top lists of tyrosine kinases (PTK) and serine/threonine (STK) kinases identified in CTC‐ITB‐01 suspension versus adherent cell fraction. The arrows indicate the kinases that have been assessed in western blot analysis.

Western blot analysis of pSRC family Y416 confirmed the upregulation of these kinases in adherent cells compared to suspension cells (*p* = 0.0353). Regarding the serine/threonine kinases, the upregulation of pPKG2 S126 (*p* = 0.0368) and pMSK1 S376 (*p* = 0.0281) was confirmed in adherent cells (Figure 6).

**FIGURE 7:**
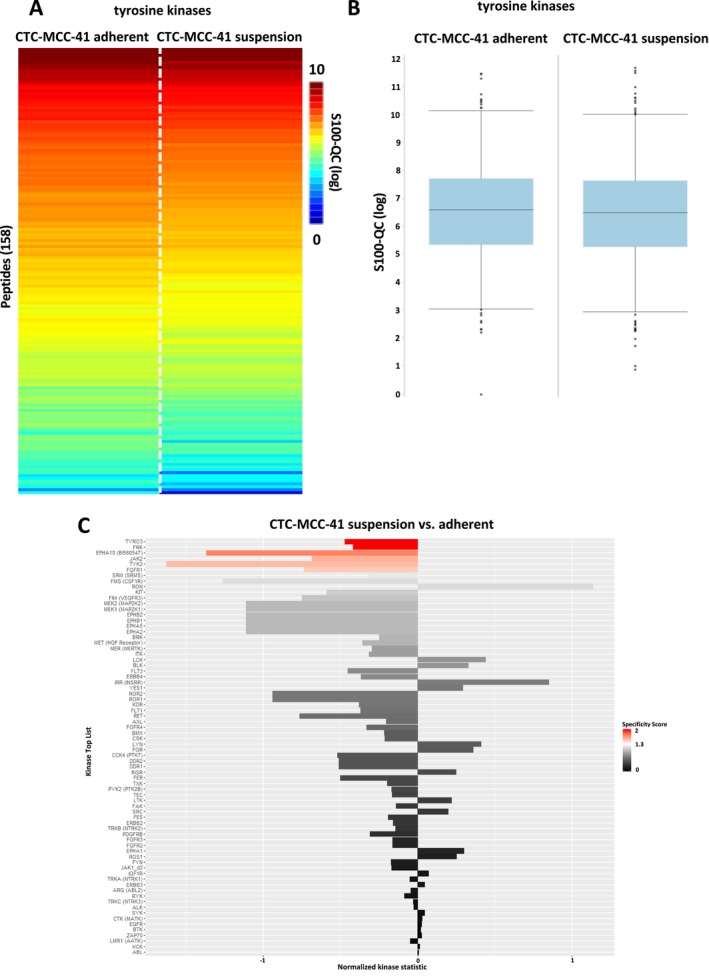
Tyrosine kinase profiling in CTC‐MCC‐41 suspension and adherent fraction. (A) Heatmap of mean log‐transformed S100‐QC values from tyrosine kinase activity measurement of CTC‐MCC‐41 adherent and suspension cells (*n* = 3 each group). (B) Boxplots of mean log‐transformed S100‐QC values (*n* = 3 each group) from tyrosine kinase analysis of CTC‐MCC‐41 adherent and suspension cells. (C) Predicted upstream tyrosine kinase list based on the results from the PamGene array (196 tyrosine peptides). Significant regulated upstream kinases were color coded based on their specificity score (white [1.3] to red [2]). Non‐significant upstream kinases are colored in a gray to black gradient.

Regarding the colorectal cancer CTC‐MCC‐41 line, again a high similarity between adherent and suspension cells was detected (Figure 7A,B). Analysis of tyrosine kinases indicated a significant upregulation of six kinases including TYRO3, FRK, EPHA10, JAK2, TYK2, and FGFR1 in the adherent fraction of CTC‐MCC‐41 (Figure 7C).

**FIGURE 8:**
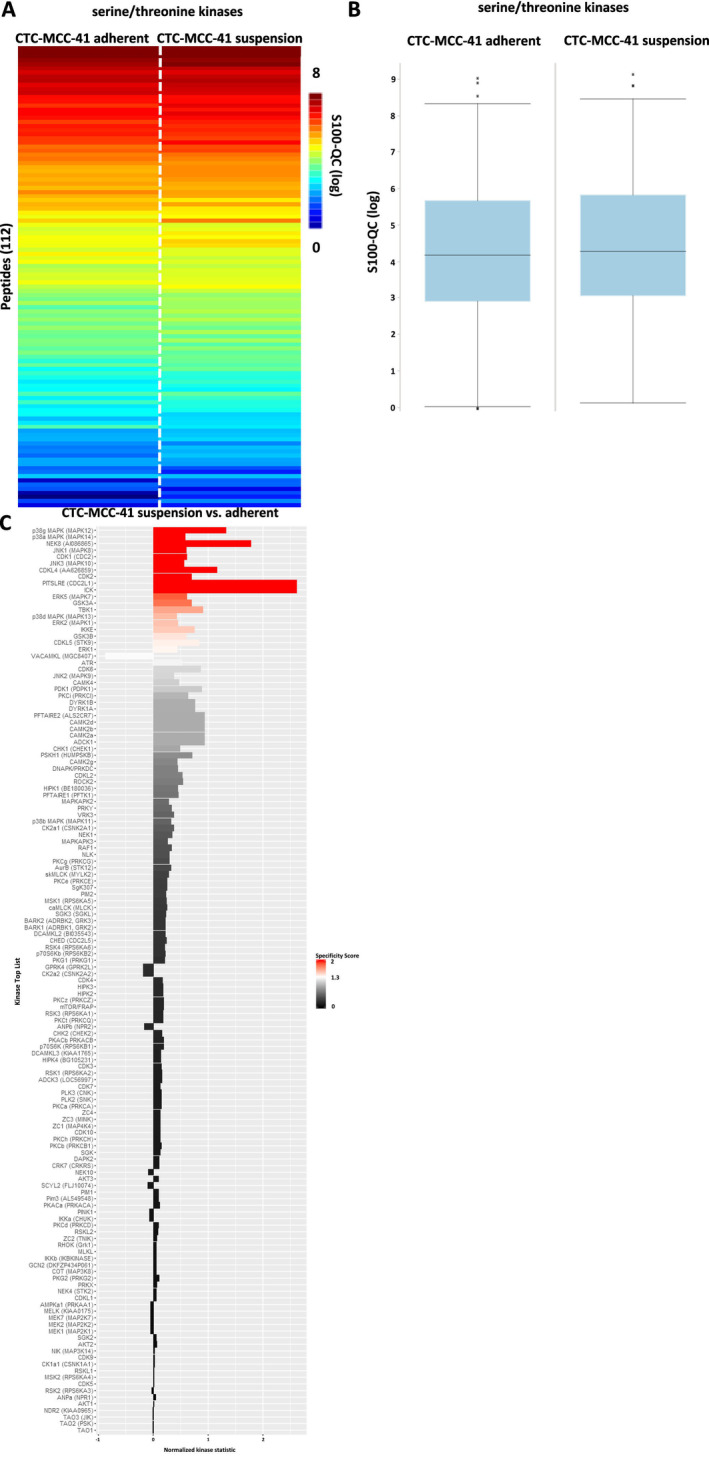
Serine/threonine kinase profiling in CTC‐MCC‐41 suspension and adherent fraction. (A) Heatmap of mean log‐transformed S100‐QC values from serine/threonine kinase activity measurement of CTC‐MCC‐41 adherent and suspension cells (*n* = 3 each group). (B) Boxplots of mean log‐transformed S100‐QC values (*n* = 3 each group) from serine/threonine kinase analysis of CTC‐MCC‐41 adherent and suspension cells. (C) Predicted upstream serine/threonine kinase list based on the results from the PamGene array (144 serine/threonine peptides). Significant regulated upstream kinases were color coded based on their specificity score (white [1.3] to red [2]). Non‐significant upstream kinases are colored in a gray to black gradient.

Interestingly, a downregulation of several serine/threonine kinases was identified in the adherent fraction of CTC‐MCC‐41 (Figure 8A–C). Among the significantly regulated kinases, several isoforms of the p38 MAPK and JNK were detected as downregulated in adherent cells (Figure 8C).

**FIGURE 9:**
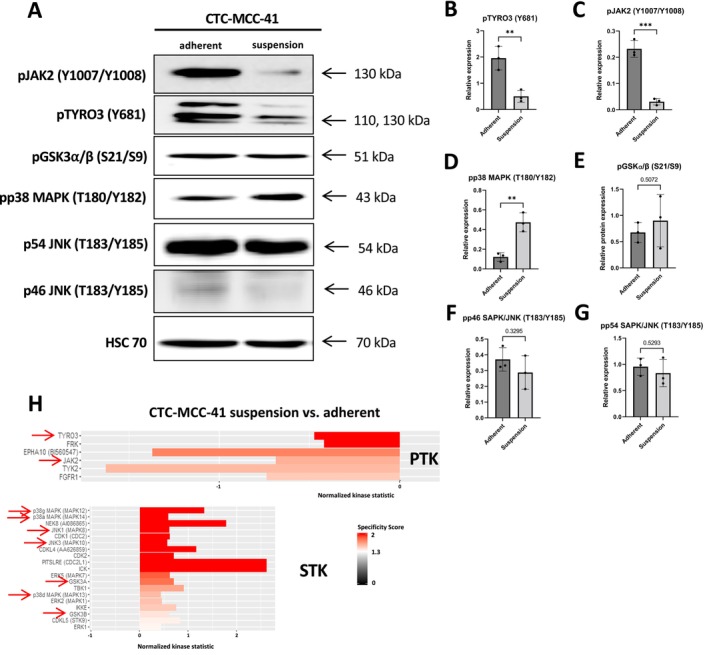
Confirmation of identified kinases in CTC‐MCC‐41 cells by western blot analysis. (A) Western blot analysis of predicted upstream kinases by the PamGene arrays in CTC‐MCC‐41 adherent and suspension cells fraction. The expression of pTYRO3 (Y681) (B), pJAK2 (Y1007/Y1008) (C), pp38 MAPK (T180/Y182) (D), pGSK3α/β (S21/S9) (E), pp46 SAPK/JNK (T183/Y185) (F), and pp54 SAPK/JNK (T183/Y185) (G) was used to validate the results from the kinome profiling (*n* = 3, unpaired two‐sided *t*‐test, **p ≤ 0.01, ***p ≤ 0.001, mean values with standard deviation). (H) Excerpt from the kinase top lists of tyrosine kinases (PTK) and serine/threonine (STK) kinases identified in CTC‐MCC‐41 suspension versus adherent cell fraction. The arrows indicate the kinases that have been assessed in western blot analysis.

Western blot analysis confirmed the upregulation of pTYRO3 Y681 (*p* = 0.0076), pJAK2 Y1007/Y1008 (*p* = 0.0005) and the downregulation of pp38 MAPK T180/Y182 (*p* = 0.0045) in CTC‐MCC‐41 adherent cells. Regarding the regulation of pGSK3α/β S21/S9 (*p* = 0.5072), pp46 SAPK/JNK T183/Y185 (*p* = 0.3295) and pp54 SAPK/JNK T183/Y185 (*p* = 0.5293) no significant differences were observed between the two fractions of CTC‐MCC‐41 in the western blot analysis (Figure 9).

## 4 Discussion

Circulating tumor cells (CTCs) have been a topic of interest since more than 50 years, but just recently subjected to extensive characterization since commercial methods for isolation became available. Interestingly, among the reported permanent cell lines, a vast amount of cell lines isolated from the blood exhibited a mixed suspension/adherent phenotype in the 2D cell culture. These cell lines that feature this phenotype include the here investigated CTC‐MCC‐41 and the CTC‐ITB‐01 cells but also other CTC lines including the CTC‐UWG‐01 and CTC‐UWG‐02 lines [10]. This behavior may indicate a high plasticity of the CTCs. An explanatory model may be that the suspension cells represent the cells that have undergone EMT, necessary for the detachment from the primary tumor. On the other hand, the adherent cells may represent the reverse EMT fraction, also referred to as MET, that allows implantation of the cells at the secondary side for metastasis formation. In this work, experiments involving only one of the fractions (i.e., adherent and suspension cells) demonstrate the reversal capacity of these cells. Surprisingly, especially suspension cells showed a high degree of plasticity to give rise to the respective adherent counterpart in the 2D cell culture. Additionally, cell counting assays revealed that the CTCs have the capability to approximately restore the initial proportion of suspension and adherent cells in the cell culture which may indicate a steady state like condition programmed within these cells. However, cultivation of only one fraction did not ultimately lead to the same proportion of cells compared to the standard culture condition state. One potential explanation for this may be a confluence dependent arrangement of cell fractions due to secreted factors or pH changes that contribute to attachment and detachment. For instance, the CTC‐ITB‐01 cells tend to form large cell cluster in suspension at higher cell confluence. On the contrary, lower confluence reverses the large cell clusters and forces the cells into a more adherent phenotype again. Under no circumstances, a monoculture of either suspension or adherent cells was possible in CTC‐MCC‐41 or CTC‐ITB‐01 cells when cultivating in 2D cell culture. To find mechanistic explanation and to further characterize the cells, signaling pathway analysis was conducted. Interestingly, the CTC‐MCC‐41 cell line showed a higher expression of pAKT S473 in the adherent cells, but not of other key molecules within the PI3K/AKT/mTOR pathway or RAS/RAF/MEK/MAPK pathway. The CTC‐ITB‐01 line did not show an alteration in pAKT S473, but rather an upregulation of pERK1/2 T202/Y204 expression in its adherent cell fraction.

The downregulation of pAKT S473 has been reported in the past after induction of EMT in lung cancer cells [14] which may support the hypothesis of adherent and suspension cells mimicking the epithelial and mesenchymal phenotype. Moreover, the downregulation of a crucial cellular pathway may implicate a quiescent like state of the suspension CTCs circulating in the blood stream compared to the adherent counterpart [15]. The observed downregulation of pERK1/2 T202/Y204 in the suspension fraction of CTC‐ITB‐01 cells is in line with the previous reports, as the p44/p42 MAPK has been described to promote EMT in different cancer entities [16, 17].

As the analysis of single hand‐picked proteins is not efficient and the expression of phosphorylated proteins may not be linked to activity, functional kinome profiling was performed on the suspension and adherent fraction of both CTC lines. The upstream kinase prediction revealed an upregulation of Src family kinases, MSK1 and PKG within the CTC‐ITB‐01 adherent fraction (compared to the suspension fraction). Interestingly, recent research revealed that capillary retention of CTCs mediated by microtentacle formation [18] was significantly increased following inhibition of Src activity [19]. Similarly, the low expression of MSK1, which was downregulated in the suspension cell fraction of CTC‐ITB‐01, was shown to regulate metastatic dormancy and be associated with early metastasis in ER^+^ breast cancer [20]. Moreover, regarding the role of PKG in cancer, it has been demonstrated that the activation of PKG is sufficient to induce apoptosis and to inhibit cell migration of colorectal cancer cells [21]. Regarding the observed lower activity of the PKG as predicted by kinome profiling this may implicate a higher metastatic potential and survival benefit of the CTC‐ITB‐01 suspension fraction. The apoptotic effect of PKG activation by cyclic GMP has also been reported elsewhere in ER^+^ and ER^−^ breast cancer cell lines [22]. Taken together, the previous reported data and the acquired data in this work may indicate a survival benefit of CTC‐ITB‐01 suspension cells, which also accounts for the largest proportions in these cells in the cell culture.

Functional kinome analysis of the CTC‐MCC‐41 colorectal CTC line revealed an upregulation of tyrosine kinases TYRO3 and JAK2 in adherent cells. TYRO3 is a member of the TAM receptor family that has not been well studied yet. However, regarding expression and activation of TYRO3 a pro‐tumorigenic role in several cancer entities including colorectal cancer has been reported. Most interestingly, it has been shown that TYRO3 signaling activates PI3K/AKT pathway mediated downstream signaling [23], which may be responsible for the higher expression of pAKT S473 in the adherent fraction of CTC‐MCC‐41 that showed an upregulation of TYRO3 activity in the functional kinome analysis. Nevertheless, previous publication reported that downregulation of TYRO3 by siRNA mediated knockdown was able to prevent melanoma cell migration and invasion [24] which would not fit into the proposed model of metastatic suspension cells in CTCs. Another interesting fact that was revealed by the functional kinome profiling, was the downregulation of p38 MAPK pathway in adherent cells compared to the suspension fraction. p38 MAPKs respond to cellular stress stimuli including cytokines, osmotic stress, hypoxia as well as shear stress [25, 26, 27], which would be in line with the suspension properties. However, in this work no alternation of SAPK/JNK related signaling that was previously described for p38 MAPK activation could be detected in the CTC‐MCC‐41 cells [28]. The fact that no overlap in the western blot signaling pathway analysis as well as the functional kinome profiling was detected underlines the high heterogeneity CTCs derived from different tumor entities, as well as the high degree of heterogeneity within different patient tumors [29].

Interestingly and in line with the previous report on AST, the switch of the cadherin expression signature that can be used to characterize EMT [30] does not seem to play a role in the anchorage dependent reprogramming [12]. The CTC‐MCC‐41 line was characterized as an intermediate epithelial‐mesenchymal line due to its expression of EpCAM, CK19, E‐cadherin and the absence of vimentin while expressing EMT inducer SNAIL [6]. The same applies to the CTC‐ITB‐01 cell line, as Koch et al. [9] reported epithelial, as well as mesenchymal traits in this cell line. Objective assessment using an EMT score, which was based on RNA‐sequencing data, revealed that the CTC‐ITB‐01 line falls more on the epithelial end of the spectrum [9].

Although our study provides valuable insights into the plasticity of CTCs and its regulation by kinases, some limitations are present as our study was conducted under 2D adherent cell culture conditions. First, in order to maintain the culture of the CTC lines, cell culture media with an unphysiological amount of glucose and higher nutrient supply is used, that does not reflect the limited nutrient supply CTCs face in the blood and may affect the tightly orchestrated signaling pathways in the cells. In addition to the lack of nutrient limitation, 2D cell culture systems do not account for fluid shear stress, hypoxia and cell–cell interactions (i.e., with other host cells) the CTCs may encounter in the blood. These limitations could be partly overcome in perfused 2D models as demonstrated earlier [31] or using starvation media to mimic the limited nutrient supply under physiological conditions. Despite the molecular characterization of the CTC lines in previous work as well as in this work, little is known about the biological relevance of the two fractions. The phenomenon of suspension and adherent cells in one CTC line has been reported in different cancer entities by different authors, yet not understood. However, the impossibility to create monocultures of adherent or suspension cells in the CTC‐MCC‐41 or CTC‐ITB‐01 cell line and the capability to give rise to the counterpart indicates a high importance of these features which should be further evaluated in the future.

## Author Contributions

**Daniel J. Smit:** conceptualization (equal), data curation (equal), formal analysis (equal), funding acquisition (equal), investigation (equal), methodology (equal), project administration (equal), resources (equal), software (equal), supervision (equal), validation (equal), visualization (equal), writing – original draft (equal), writing – review and editing (equal). **Konstantin Hoffer:** formal analysis (equal), investigation (equal), software (equal), visualization (equal), writing – review and editing (equal). **Bettina Bettin:** data curation (equal), investigation (equal), writing – review and editing (equal). **Malte Kriegs:** formal analysis (equal), methodology (equal), resources (equal), writing – review and editing (equal). **Laure Cayrefourcq:** resources (equal), writing – review and editing (equal). **Udo Schumacher:** conceptualization (equal), methodology (equal), supervision (equal), writing – review and editing (equal). **Klaus Pantel:** conceptualization (equal), methodology (equal), resources (equal), supervision (equal), writing – review and editing (equal). **Catherine Alix‐Panabières:** conceptualization (equal), methodology (equal), writing – review and editing (equal). **Manfred Jücker:** conceptualization (equal), formal analysis (equal), funding acquisition (equal), methodology (equal), resources (equal), supervision (equal), validation (equal), writing – review and editing (equal).

## Conflicts of Interest

The authors declare no conflicts of interest.

## Supporting information


Table S1.

## Data Availability

Data available within the article or in the Supporting Information.
