# Correction to “Analysis of the Plasticity of Circulating Tumor Cells Reveals Differentially Regulated Kinases During the Suspension‐to‐Adherent Transition”Smit, D., Hoffer, K., Bettin, B., Kriegs, M., Cayrefourcq, L., Schumacher, U., Pantel, K., Alix‐Panabières, C. and Jücker, M. (2024), Analysis of the Plasticity of Circulating Tumor Cells Reveals Differentially Regulated Kinases During the Suspension‐to‐Adherent Transition. Cancer Medicine, 13: e70339. https://doi.org/10.1002/cam4.70339

**DOI:** 10.1002/cam4.70442

**Published:** 2024-12-09

The funding information provided by the authors was incorrectly processed during the production of the final article. The correct funding statement is “This work was supported by the Erich and Gertrud Roggenbuck Stiftung, the National Cancer Institute (INCa) and the Open Access Publication Fund of UKE—Universitätsklinikum Hamburg‐Eppendorf.”

We apologize for this error.

