# Supplementary material for: Analysis of the Plasticity of Circulating Tumor Cells Reveals Differentially Regulated Kinases During the Suspension‐to‐Adherent Transition
Source: Cancer Med. 2024 Oct 18;13(20):e70339. doi: 10.1002/cam4.70339 (PMC11489281; doi:10.1002/cam4.70339)
Supplement: Supplementary file 1 — Table S1. [file CAM4-13-e70339-s001.docx]

**11. SUPPLEMENTAL MATERIAL**

**Supplemental Table 1:** Antibodies used in the study.

| **Target** | **Manufacturer** | **Clone / Reference number** | **Secondary antibody** | **Dilution** |
| --- | --- | --- | --- | --- |
| Anti-mouse IgG, HRP-linked | Cell Signaling Technology | #7076 | - | 1:5000 |
| Anti-rabbit IgG, HRP-linked | Cell Signaling Technology | #7074 | - | 1:5000 |
| HSC70 | Santa Cruz Biotechnology | B-6, #sc-7298 | mouse | 1:1000 |
| mTOR | Cell Signaling Technology | 7C10, #2983 | rabbit | 1:1000 |
| pAxl (Y698)/ pMer (Y749)/pTyro3 (Y681) | Cell Signaling Technology | D6M4W, #44463 | rabbit | 1:1000 |
| p44/42 MAPK (Erk1/2) | Cell Signaling Technology | #9102 | rabbit | 1:1000 |
| pAKT (S473) | Cell Signaling Technology | D9E, #4060 | rabbit | 1:1000 |
| panAKT | Cell Signaling Technology | 11E7, #4685 | rabbit | 1:1000 |
| pGSK-3α/β (S21/S9) | Cell Signaling Technology | 37F11, #9327 | rabbit | 1:1000 |
| pJAK2 (Y1007/Y1008) | Cell Signaling Technology | #3771 | rabbit | 1:1000 |
| pMSK1 (S376) | Cell Signaling Technology | #9591 | rabbit | 1:1000 |
| pmTOR (S2448) | Cell Signaling Technology | #2971 | rabbit | 1:1000 |
| pp38 MAPK (T180/Y182) | Cell Signaling Technology | #9211 | rabbit | 1:1000 |
| pp44/42 MAPK (Erk1/2) (T202/Y204) | Cell Signaling Technology | #9101 | rabbit | 1:1000 |
| pPKG2 (S126) | Biorbyt | #orb335852 | rabbit | 1:1000 |
| pS6 ribosomal protein (S240/S244) | Cell Signaling Technology | D68F8, #5364 | rabbit | 1:1000 |
| pSAPK/JNK (T183/Y185) | Cell Signaling Technology | #9251 | rabbit | 1:1000 |
| pSrc Family (Y416) | Cell Signaling Technology | D49G4, #6943 | rabbit | 1:1000 |
| S6 ribosomal protein | Cell Signaling Technology | 5G10, #2217 | rabbit | 1:1000 |
